# A controlled double-duration inducible gene expression system for cartilage tissue engineering

**DOI:** 10.1038/srep26617

**Published:** 2016-05-25

**Authors:** Ying Ma, Junxiang Li, Yi Yao, Daixu Wei, Rui Wang, Qiong Wu

**Affiliations:** 1 MOE Key Laboratory of Bioinformatics, Center for Synthetic and Systems Biology, Tsinghua University, Beijing, China; 2 School of Life Sciences, Tsinghua University, Beijing, China; 3 Center for Synthetic & System Biology, Tsinghua University, Beijing, China

## Abstract

Cartilage engineering that combines competent seeding cells and a compatible scaffold is increasingly gaining popularity and is potentially useful for the treatment of various bone and cartilage diseases. Intensive efforts have been made by researchers to improve the viability and functionality of seeding cells of engineered constructs that are implanted into damaged cartilage. Here, we designed an integrative system combining gene engineering and the controlled-release concept to solve the problems of both seeding cell viability and functionality through precisely regulating the anti-apoptotic gene bcl-2 in the short-term and the chondrogenic master regulator Sox9 in the long-term. Both *in vitro* and *in vivo* experiments demonstrated that our system enhances the cell viability and chondrogenic effects of the engineered scaffold after introduction of the system while restricting anti-apoptotic gene expression to only the early stage, thereby preventing potential oncogenic and overdose effects. Our system was designed to be modular and can also be readily adapted to other tissue engineering applications with minor modification.

Tissue engineering is an interdisciplinary science requiring a diligent combination of suitable biomaterials and seeding cells to gain improved functionality during the regeneration process^1^. Judging from previous studies, successful engineering requires both the maintenance of cell viability and the production of functional tissue. Both areas are studied intensively by researchers using different methods^2^,^3^,^4^,^5^,^6^.

Significant reduction of cell viability is a severe problem not only for cartilage tissue engineering but also for nearly all applications of engineered implants. Basic biological studies have demonstrated that apoptosis and necrosis can be quickly induced after transplantation due to the transition from the *in vitro* growth factor-supplied culture medium environment to one of compromised nutrition and abundance of apoptotic signal molecules at the damaged site^7^. An additional source of stress for the transplanted cells is the attack by the innate immune system^8^. Studies have applied modified scaffolds to enhance cell attachment^9^, and in some cases, harbored growth factors to support seeding cell viability in the early stage^10^,^11^. Other studies also explored the possibility to use gene engineering approaches to overexpress anti-apoptotic genes in seeding cells^12^,^13^. However, although the expression of anti-apoptotic genes was shown to effectively improve the overall cell viability, sustained expression of these genes is associated with oncogenic effects^14^,^15^, which severely hampers practical application.

The functionality of the seeded cells is critical for achieving satisfactory final regenerative outcomes. In cartilage engineering, *in vitro* expanded mesenchymal stem cell (MSCs) are the most commonly used seeding cell type because MSCs can be induced to undergo chondrogenesis both *in vitro* and *in vivo*, thus gradually filling and functionalizing the site of cartilage damage^14^,^15^. However, in therapeutic applications, pre-induction of chondrogenesis in the MSCs was mainly achieved by complex chemical or growth factor supplements with low efficiency and high cost^16^,^17^. Alternatively, direct regulation of the expression of key transcriptional factors in seeding cells is considered to be a direct and specific strategy in both bone and cartilage tissue engineering^18^,^19^. For instance, our group has successfully inhibited the *in vitro* degeneration of primary chondrocytes through controlled expression of sox9, a key chondrogenesis regulator, to sustain significant chondrocyte functionality even after extended *in vitro* culture^20^.

In the present study, we focused on designing an integrative controlled platform harboring genetically engineered MSCs to both improve cell viability and enhance chondrogenic performance via precise controls. Gene engineered MSC cell lines were constructed comprising two independent inducible gene expression modules, expressing the anti-apoptotic bcl-2 gene and the chondrogenic master regulatory sox9. Two non-toxic small molecules for specific induction were assembled in two controlled-release modules (a direct absorption module and a microsphere encapsulation module) conjugated with the gelatin sponge scaffold to achieve controlled-duration gene expression. With intensive optimization of the modules in this system, the anti-apoptotic effect of Bcl-2 could be restrictively activated within the first week upon implantation, while relatively long-term expression of Sox9 was executed to facilitate chondrogenic commitment during the first 3–4 weeks after implantation. Our system better mimics the *in vivo* situation and succeeds in this way not only in achieving improved cell cellularity and chondrogenic matrix secretion but also in preventing the potential oncogenic and overdose effects of regulatory genes due to persistent activation under traditional constituent promoter systems. As our basic design followed the module concept, our platform is readily adaptable to other tissue engineering applications.

## Materials and Methods

### Cell lines and culture media

The C3H10T1/2 murine mesenchymal stem cell line was cultured in MEM/EBSS medium (HyClone, Thermo Scientific, USA) supplemented with 10% fetal bovine serum (FBS, Sigma Dorset, UK) and 100 μg/mL of penicillin–streptomycin (Inalco, USA). All cells were cultured at 37 °C in a humidified incubator with a mixture of 5% CO_2_ and 95% air. For the *in vitro* evaluation of cell status in the overall engineered system with controlled release of both doxycycline and coumermycin, 50% of the culture media was changed every day to mimic, to a certain extent, the *in vivo* fluidic environment.

### Plasmid construction and transfection

Plasmids used in this study comprise a tet-on inducible system and a regulated mammalian coumermycin inducible expression system. EGFP-N1 (Clonetech, USA) was used as a control. The tet-on inducible system was as previously described^20^. Sox9 expression was induced with the tet-on system, and the two components of this system (PTE and RT-TA) were integrated into the seeding cells via lentivirus infection for long-term expression. The coumermycin inducible system (Promega, USA) was used for short-term expression of Bcl-2; this regulated mammalian expression system was delivered as two components into C3H10T1/2 cells by electroporation using the T-020 program (Amaxa, Biosystems, Germany). The details of the plasmids used in this study are listed in Supplemental Table S1.

### Components and general workflow for establishing a controlled double-duration inducible gene expression system

**Figure 1:**
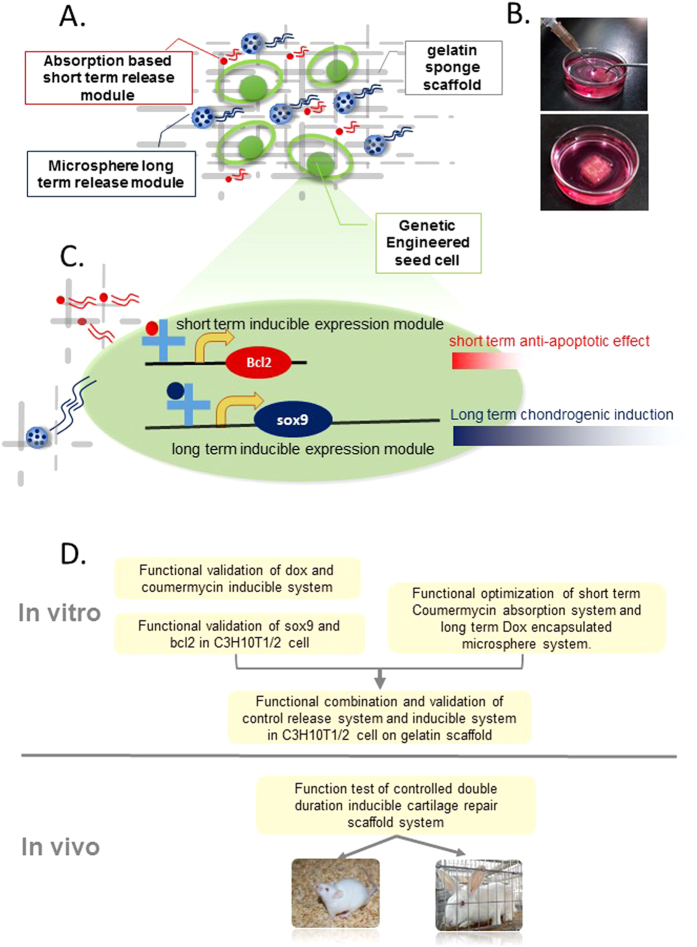
Components and general workflow for establishing a controlled double-duration inducible gene expression system. (**A**) Schematic diagram of our whole experimental design, which comprises engineered seed cells (green ellipses), a gelatin sponge scaffold and two different release systems for indicated chemicals: doxycycline (dark blue particles in the larger blue microspheres) and coumermycin (red particles) to control their long-term and short-term release, respectively. (**B**) Snapshot of injection of seed cells and controlled release modules into the gelatin sponge scaffold (up); snapshot of *in vitro* culture of controlled double duration inducible cartilage repair scaffold system (bottom). (**C**) General mode for duration based dual-switch system in seed cells; (**D**) The whole workflow of our experiment, which consisted of *in vitro* and *in vivo* experiments.

To achieve both viability and real functionality of chondrogenic seeding cells, we sought to design a cartilage gelatin sponge scaffold containing two non-toxic small molecules for controlled release with different duration. Coumermycin, which is directly adsorbed onto the gelatin sponge scaffold, was used for short-term induction of the viability maintenance (anti-apoptotic) module. Dox (doxycycline) was encapsulated in the PLGA microspheres for long-term release and was used to induce chondrogenic differentiation (Fig. 1A,B).

In the engineered seeding cells, the short-term inducible expression module consisted of a coumermycin-inducible Bcl-2 gene expression construct (Fig. 1C, top). As the expression of Bcl-2 was designed to contribute to the anti-apoptotic effect within the defined short time interval, coumermycin was provided at the early stage of implantation with a quickly decreasing concentration to prevent the potential long-term danger of cancerous changes (Fig. 1C, top). In parallel, relatively long-term chondrogenic induction of seeding cells was directed by the chondrogenic master transcriptional factor Sox9, which was controlled by a dox inducible expression construct. The long-term controlled-release nature of PLGA microspheres harbored dox-enabled sustained expression of Sox9, which in turn directed long-term chondrogenic commitment (Fig. 1C, bottom).

We first tested and confirmed the rational design of our system *in vitro* to optimize the dose and the duration of the release of the inducing chemicals (Fig. 1D, Top). We next tested the effectiveness of this system in both a mouse transplantation model and a rabbit cartilage repair model (Fig. 1D, bottom).

### Real-time polymerase chain reaction (RT-PCR)

Total RNA was isolated from C3H10T1/2 cells using a miRcute mRNA Isolation Kit (Tiangen, Beijing, China). cDNA was synthesized using a Fastquant RT Kit (Tiangen, Beijing) for mRNA analysis. qRT-PCR was performed with SuperReal PreMix (SYBRGreen) (Tiangen, Beijing), and GAPDH was used as an internal control. The primers used for RT-PCR are listed in Supplemental Table S2.

### Determination of cell survival rates

Cell survival rates were assessed using a CCK8 Cell Counting Kit (Beyotime, China), according to the manufacturer’s instructions. Cells were either transfected with the BCL2 overexpression plasmid or with EGFP-N1 as a control (Table S1). Cells were seeded in flat-bottomed 96-well plates at 1 × 10^4^ cells/well and incubated in MEM/EBSS (HyClone, Thermo scientific, USA) medium containing 10% FBS for 24 hours in 37 °C in a humidified incubator with a mixture of 5% CO_2_ and 95% air. Each group comprised 8 wells. Subsequently, the cells were transferred to MEM/EBSS medium without FBS and cultured for an additional 24 hours to induce apoptosis. A total of 10 μL of CCK-8 was added to each well, and 100 μl of MEM/EBSS containing 10% FBS and antibiotic without any CCK8 was used as photometric blank. After incubation at 37 °C for 2 hours, the absorbance at 450 nm of each well was determined using a microplate reader (Molecular Devices, USA), and the survival rate was calculated according to the CCK8 kit instructions.

### Creation of controlled-release modules by microsphere encapsulation and direct absorption

Dox (doxycycline) was encapsulated in the poly(lactic-co-glycolic acid (PLGA) microspheres for long-term release and was used to induce chondrogenic differentiation (Fig. 1A). PLGA microspheres were prepared using the improved W/O/W method^21^. In particular, 1 mg of PLGA ((Mw = 9000/20000/45000 Da, Daigang, China) was dissolved in 20 ml of dichloromethane to form a PLGA oil phase solution (O). The first water phase (W1) dox solution (20 mg/mL doxycycline, Sigma, USA) and the PLGA oil phase solution were mixed by Ultra-Turrax (IKA®T10 B S25, Germany) at a gear range of 6 for 30 s to form the first water-in-oil (W1/O) emulsion. The first emulsion was added to 20 ml of a 1% (w/v) polyvinyl alcohol (PVA) second water phase solution (W2) and then stirred at 400 rpm for 12 h to form the final emulsion (W1/O/W2), drive off the dichloromethane and solidify the microspheres. The microspheres were collected after sedimentation, washed with PBS buffer, and stored at 4 °C for later use. Coumermycin (Promega, USA) was directly absorbed onto the gelatin sponge (Nanjing Jinling pharmaceutical factory, China) by injecting it as a 5-mM solution in DMSO diluted in ethanol to 20-μM and then air dried to evaporate the ethanol.

### Animal model experiments

All animal experiments were approved by *the Institutional Animal Care and Use Committee of Tsinghua University*. Animal care and experimental procedures were carried out in accordance with *the guidelines of the Institutional Animal Care and Use Committee of Tsinghua University* and *the National Institutes of Health Guide for the Care and Use of Laboratory Animals.* The engineered cell-containing scaffolds were prepared *in vitro* before implantation. They comprised the absorbable gelatin sponge (Nanjing Jinling pharmaceutical factory, China), the PLGA microspheres to release dox, the coumermycin absorbed onto the gelatin sponge and the C3H10T1/2 cells containing the two gene inducible expression constructs. C3H10T1/2 cells transfected with EGFP-N1 and without any inducer used as a control. The experimental treatments were C3H10T1/2 transfected with the coumermycin-inducible system and with only dox induction (released by PLGA microspheres), C3H10T1/2 transfected with coumermycin-inducible system and with dox (released by PLGA microsphere) and coumermycin induction (released directly by the absorbable gelatin sponge).

For the subcutaneous implantation model, 5-week-old BALB/c mice (vital river, China) were used. The mice were anesthetized with 1 ml/g body weight of 4% pentobarbital sodium in saline via a peritoneal injection. Procedure details were as described previously^22^. Two implants with the same indicated treatment were implanted for each mouse at the left and right side of the back. Two batches of mice were sacrificed after 1 week and 4 weeks for RT-PCR, hematoxylin and eosin (HE) staining and alcian blue staining. Numbers of subcutaneous implants for each experimental conditions are summarized below (Table 1).

For the osteochondral restoration model, 3-month-old New Zealand rabbits (vital river, China) were used (body weight: 2.7 ~ 3.0 kg). The rabbits were anesthetized with 1 ml/kg body weight 4% pentobarbital sodium in saline via an auricular vein injection. Full thickness cartilage defects were created surgically on the femoropatellar groove of the knee joints and extended though the cartilage layer, penetrating the subchondral bone. The defects were 4 mm in diameter and 2 mm in depth and were filled with the engineered cell-containing scaffolds using the press-fit method as described in our previous paper^23^. Two full thickness cartilage defects were created surgically on the femoropatellar groove of the knee joints with the same indicated treatment at the right leg of the rabbit (no surgery at the left leg of the rabbit). The rabbits were sacrificed after 30 days, the defects with transplanted samples were removed and decalcified using EDTA decalcifying fluid (Solarbio, China) for 10 days at 37 °C, after which they were embedded in paraffin, sectioned (5 μm) and observed histologically using HE staining and alcian blue staining. Numbers of cartilage defects for each experimental conditions are summarized below (Table 2).

### HE staining, alcian blue staining and immunohistochemistry

Samples were fixed in 4% paraformaldehyde (Dingguo Changsheng Biotechnology Co. Ltd., Beijing, China) at room temperature for one day, embedded in paraffin, sectioned (5 μm), and incubated with HE (Beyotime, China) for 2 min or 8*Alcian blue (Cyagen, Guangdong, China) for 30 min. The excess stain was washed away with double-distilled water. All images were captured using a light microscope (Nikon Eclipse, Ti, Japan). For Col2A1 immunohistochemistry, goat polyclonal antibodies against mouse Col2a1 antibody (sc-52658, Santa Cruz Biotechnology, USA) was used following the manufacturer’s protocol.

### Measuring doxycycline and coumermycin concentration

To measure the dox (doxycycline) released from PLGA microspheres and the coumermycin released from the gelatin sponge, we used QTRAP 4500 LC/MS/MS (AB SCIEX, USA) to measure the concentrations. All samples of these two chemical inducers were measured at the same time under the same conditions. Chromatographic analysis was performed using a LC-20A HPLC system (shimadzu, Japan). An Eclipse plus C18 (RRHD 1.8 μm, 2.1 × 50 mm, Agilent, USA) column was used for doxycycline. The mobile phase consisted of acetonitrile (mobile phase B) and water (mobile phase A). A gradient elution starting with 98% mobile phase B (acetonitrile) was decreased linearly to 0% mobile phase B over 6 min, kept constant at 0% for 1 min, and then increased to 98% B in 0.1 min, and this composition was maintained until the end of the run (10 min). The flow rate was set at 0.2 ml/min. The column effluent was monitored using a 4500 TRAP^TM^ LC/MS/MS. The optimized precursor ions pairs were m/z 445.200 → 410.000 for doxycycline. The parameters were as follows: the curtain gas was 10 Psi; the temperature was maintained at 500 °C; ionspray voltage was 4500 V; and the collision gas was set at medium. For coumermycin, a Poroshell 120 SB-C18 column (2.7 μm, 2.1 × 100 mm) was used. The mobile phase consisted of acetonitrile (mobile phase B) and water (mobile phase A). A gradient elution starting with 90% mobile phase B (acetonitrile) was decreased linearly to 20% mobile phase B over 2 min, then decreased to 0% mobile phase B over 4 min, kept constant at 0% for 1 min, and then increased to 90% B in 0.1 min, and this composition was maintained until the end of the run (7 min). The flow rate was set at 0.4 ml/min. The column effluent was monitored using a 4500 TRAP^TM^ LC/MS/MS. The optimized precursor ions pairs were m/z 1110.100 → 282.000 for doxycycline. The parameters were as follows: the curtain gas was 10 Psi; the temperature was maintained at 500 °C; ionspray voltage was 5500 V; and collision gas was set at medium. Other parameters were determined for each inducer using version 1.6.1 of the Analyst software.

### Environmental SEM analysis

For environmental SEM (environmental scanning electron microscopy), the PLGA microspheres and absorbable gelatin sponge were mounted on aluminium stumps, coated with gold in a sputtering device for 1 min at 15 mA and examined under the environmental scanning electron microscope (FEI Quanta 200, Czech Republic).

### Statistical analysis

Data were expressed as the mean and standard deviation (SD) as error bar. Statistical comparisons were made between multiple groups based on a nonparametric Kruskal–Wallis (K-W) test. The significance of the results to reject the null hypothesis were calculated using IBM SPSS software. Values were considered significant at p < 0.05 or highly significant at p < 0.01.

## Results

### Inducible gene expression modules for Bcl-2 and Sox9 expression

**Figure 2:**
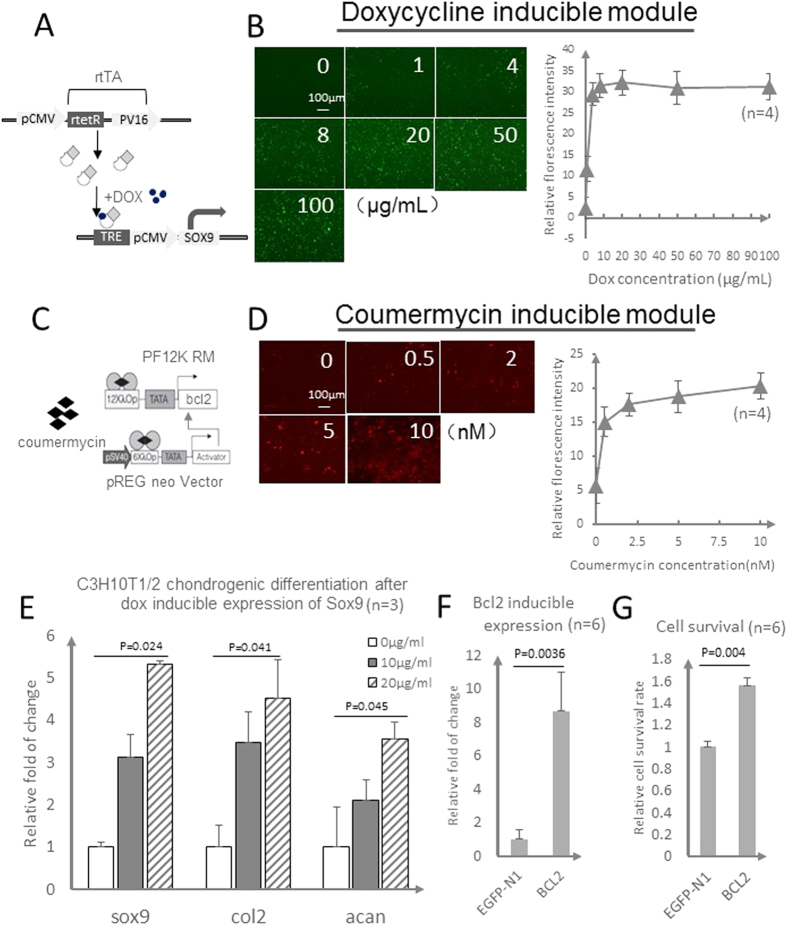
Inducible gene expression modules for Bcl-2 and Sox9 expression. (**A**–**D**) The function of doxycycline (dox) and coumermycin inducible modules. Sketches of dox (**A**) and Coumermycin (**C**) inducible modules. (**B**,**D**) The results of fluorescence and flow cytometry analyses showed the appropriate induction concentration for each system. (**E**–**G**) The function of Sox9 and Bcl2 genes in C3H10T1/2 cells using their respective appropriate concentrations to induce the two inducible systems; (**E**) Expression of Sox9 and downstream genes (Col2 and Acan) under regulation by a tet-on inducible system; n = 3. (**F**,**G**) Expression of Bcl2 and cell survival under regulation by a coumermycin inducible system; n = 6. The statistical significance was indicated by P-value.

The DOX inducible system comprises two vectors (Fig. 2A). The *RTTA vector* encodes a tetracycline inducible transcription activator that binds to the Tre sequence. It consists of a chimeric transactivator containing a VP16 transcriptional activator domain. The *Ptre Vector* contains 7 copies of the Tre sequence and a minimal CMV promoter that responds to the transactivator encoded in the RTTA vector. The chondrogenic transcriptional factor Sox9 is expressed from this vector under tetracycline induction.

The coumermycin inducible system also comprises two vectors (Fig. 2C). The *pREG neo vector* is designed to express a chimeric transactivator fusion protein comprising the VP16 transcriptional activator domain^24^ that can bind to the λ operator sequence. The *pF12K RM Flexi vector* contains tandem λ operator sequences and a minimal CMV promoter that responds to the transactivator encoded in the pREG neo vector. The well-known anti-apoptotic gene Bcl-2 is expressed from this vector.

To test the effective dose for the dox inducible system, we first ligated eGFP (enhanced green florescent protein) into this module and monitored the fluorescence intensity under a wide range of dox concentrations. The gene expression levels were positively correlated with dox concentration up to 20 μg/mL and remained stable at higher doses (Fig. 2B). Similarly, RFP (red florescence protein) expression under the coumermycin inducible system was effectively induced at concentrations as low as 2 nM (Fig. 2D).

Considering the co-transfection of multiple modules into host cells, we determined whether the two inducible systems might exhibit cross-reactivity. When the two modules were co-transfected into the seed cells, dox could only activate the long-term induction module, and coumermycin induced only the short-term expression module, implying that the modules are for all intent and purposes orthogonal (Supplemental Figure S1).

We next ligated the Sox9 gene into the dox long-term inducible module and the Bcl-2 gene into the coumermycin inducible module, assembling the integrative double-duration inducible gene expression system for the concrete cartilage engineering context. We first monitored the chondrogenic effect of the system upon dox induction. As expected, direct dox induction with concentrations of 10 μg/mL and 20 μg/mL both significantly elevated the Sox9 transcription factor expression level (Fig. 2E). The matrix proteins Collagen II (Col2) and Aggrecan (Acan), two well established markers of chondrogenesis transcriptionally controlled by Sox9^25^, were also upregulated (Fig. 2E), suggesting the feasibility of a functional dox inducible chondrogenic module. On the other hand, coumermycin at a concentration of 2.5 nM effectively induced Bcl-2 gene expression (Fig. 2F), which in turn improved the survival rate of seed cells approximately 1.5-fold (Fig. 2G). Collectively, these results demonstrated that direct dox and coumermycin induction of chondrogenic and anti-apoptotic activity was achievable using these two inducible modules.

### Modules for controlled short-term and long-term release of coumermycin and doxycycline

**Figure 3:**
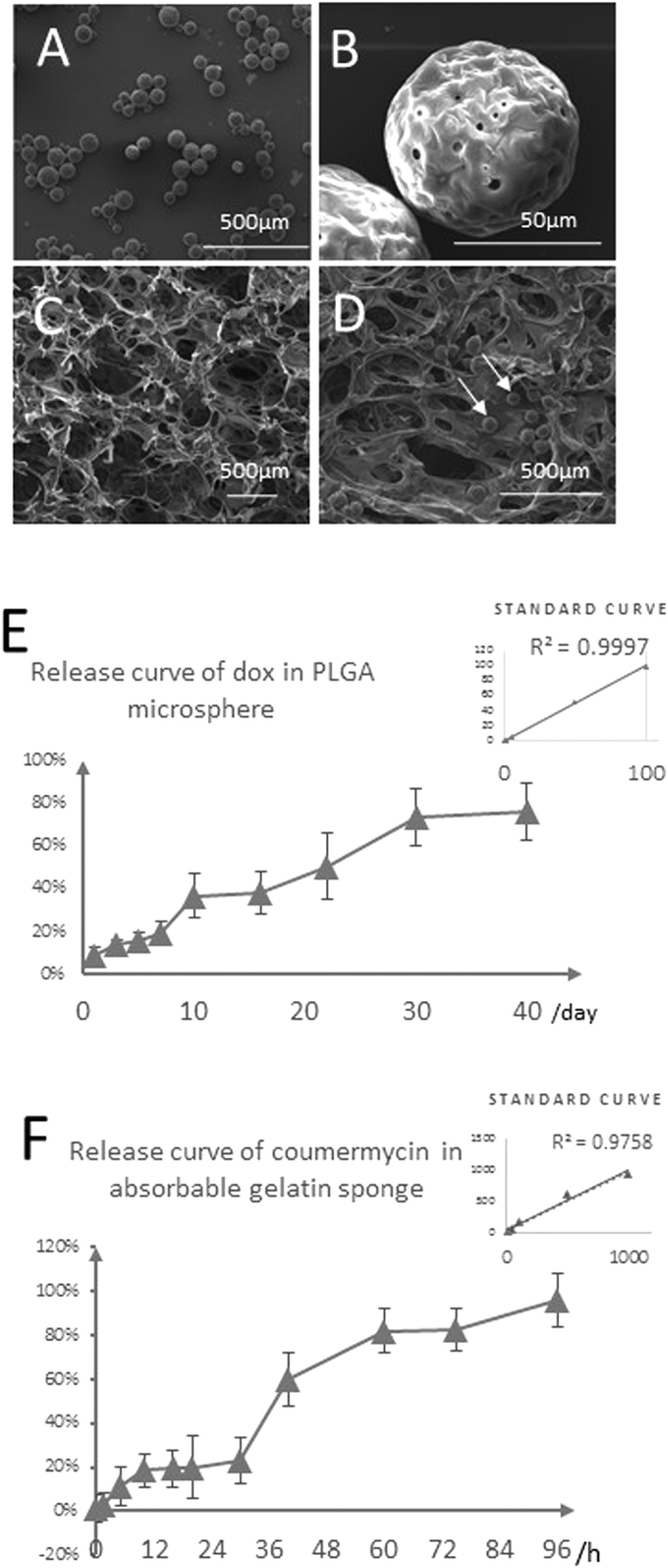
Short-term and long-term release modules for duration-controlled release of Coumermycin and Doxycyclin. (**A**–**D**) Characterization of PLGA (50/50) microspheres and the compatibility of PLGA microspheres with the absorbable gelatin sponge. White arrows indicate microspheres attached to the gelatin sponge. (**E**) The standard curve and release curve of doxycycline from PLGA microspheres *in vitro*; (**F**) The standard curve and release curve of coumermycin from absorbable gelatin sponge *in vitro*.

To achieve the long-term release of dox, we decided to apply PLGA microsphere encapsulation. PLGA microspheres have been widely studied and applied for controlled release of drugs both *in vitro* and *in vivo*^26^,^27^,^28^,^29^. After optimization of molecular weight of PLGA (MW = 9000 ~ 45000 Da), we chose MW around 20000 as to generate microspheres with the diameter mostly ranging from 70–80 μm (Fig. 3A,B), and these microspheres could be delivered into the scaffold evenly by general injection, as was visualized by SEM (Fig. 3C,D). We next established the PLGA microspheres encapsulated dox embedded in a gelatin scaffold, which was able to gradually release dox for approximately 40 days (Fig. 3E). The gradual release nature made it possible to maintain a defined working concentration under *in vitro* culture medium conditions around the scaffold. Short-term release of coumermycin was achieved by direct adsorption onto the gelatin sponge scaffold. We tested the release curve of absorption release of coumermycin and found that it was completely released within approximately 100 hours (Fig. 3F). This duration was suitable to restrict Bcl-2 expression to within ~5 days, thus preventing long-term oncogenic effects of this gene.

### Construction and validation of controlled double-duration inducible gene expression system for chondrogenesis *in vitro*.

**Figure 4:**
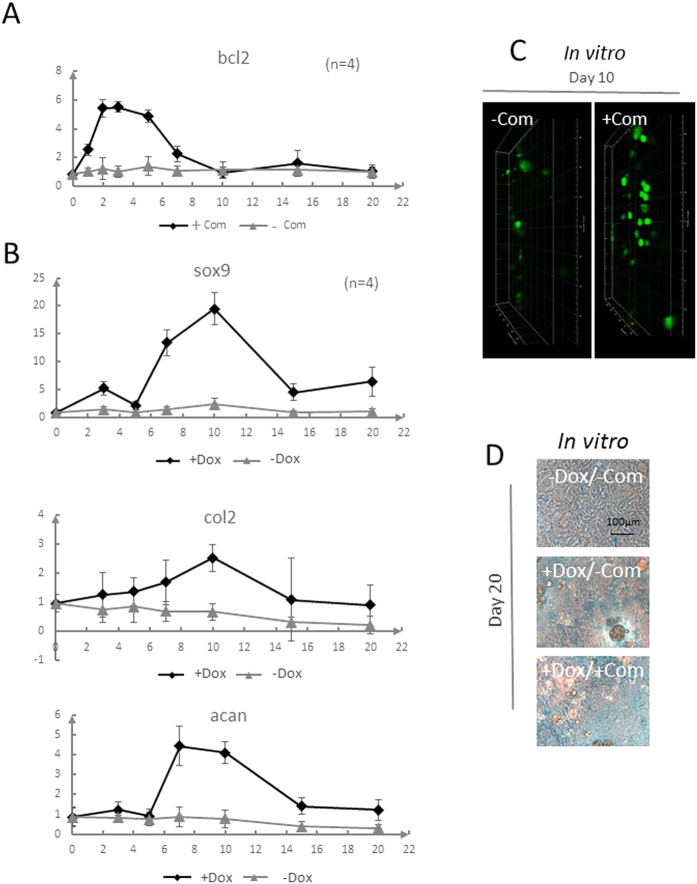
Construction and validation of controlled double duration inducible gene expression system for chondrogenesis *in vitro.* (**A**,**C**) Bcl2 expression and viable C3H10T1/2 cells in gelatin sponge scaffold under induction by the short-term inducible module. (**A**) Bcl2 expression from day 0 to day 20 for groups with and without Com induction; n = 4. (**C**) CFSE staining results illustrating difference for the cell viability at day 10 with starvation culture, revealed by multiple photon laser scanning microscope analysis. (**B**,**D**) Chondrogenesis gene expression measured via mRNA level and alcian blue staining of C3H10T1/2 cells in gelatin sponge scaffold under induction by the long-term inducible module. (**B**) Chondrogenesis gene expression measured via mRNA level during days 0 to 20; n = 4. (**D**) Alcian blue staining results on day 20. Dox: doxycycline; Com: coumermycin; CFSE: Carboxyfluorescein Succinimidyl amino Ester.

We next combined these two controlled-release modules and inducible gene expression modules to maintain cell viability at the early stage and induce sufficient chondrogenic differentiation for a relatively long interval. We used a lentiviral vector to integrate a dox inducible system for long-term expression and transiently transfected a coumermycin inducible system for short-term expression into C3H10T1/2 cells, establishing an engineered chondrogenic progenitor cell line. To evaluate the feasibility of the system *in vitro*, two controlled-release modules and cells from the engineered cell line were integrated into the gelatin sponge scaffold and cultured in the standard EBSS medium with 10% FBS in the incubator. Notably, under *in vitro* culture conditions, the adsorption-based short-term release of coumermycin successfully induced expression of Bcl-2 in C3H10T1/2 cells within 2 days, lasted for 6 days, and decreased to basal level within 10 days, demonstrating the restricted expression nature of this anti-apoptotic signal (Fig. 4A). In parallel, long-term controlled release of dox by microspheres successfully enabled Sox9 gene expression, and hence the induced expression of the matrix marker proteins Collagen II (Col2) and Aggrecan (Acan) in engineered C3H10T1/2 cells. The induced activity of these genes was monitored and remained stable for at least 20 days *in vitro* (Fig. 4B), which shows that a combination of microsphere encapsulated dox controlled release and engineered seed cells successfully achieves long-term chondrogenic induction, when compared to the control group without coumermycin induction which showed a significant chondrogenic degradation consisted with our previous study^25^. To validate the effectiveness of the overall system, we next monitored the cell viability using *in situ* CFSE (5,6- carboxyfluorescein diacetate,succinimidyl ester) staining of the gelatin sponge scaffold upon 10 days of culture with reduced serum. As expected, the engineered cells in the coumermycin induction group were markedly more viable compared with the non-induced control group from multiple photon laser microscope analysis (Fig. 4C), confirming the anti-apoptotic effects of the short-term module under *in vitro* conditions. Finally, we evaluated the acidic polysaccharide matrix secretion of the cells in the engineered scaffold using alcian blue staining. Typically, cells integrated into control scaffolds without either dox or coumermycin induction secreted only small amounts of matrix polysaccharides, and significantly more acidic polysaccharide matrix secretion was observed when the encapsulated dox microsphere long-term release module was present (Fig. 4D up and middle). When both the encapsulated dox microsphere long-term release module and the coumermycin adsorption short-term release module were integrated into the system, a further increase of the amount of the acidic matrix was observed, indicating that the improved cell viability also contributed to the overall effect of chondrogenic restoration (Fig. 4D bottom). In conclusion, we successfully assembled the controlled double-duration inducible gene expression system *in vitro* and achieved an improved restoration effect using a rational module design.

### Evaluation of our system in the mouse subcutaneous implantation model

**Figure 5:**
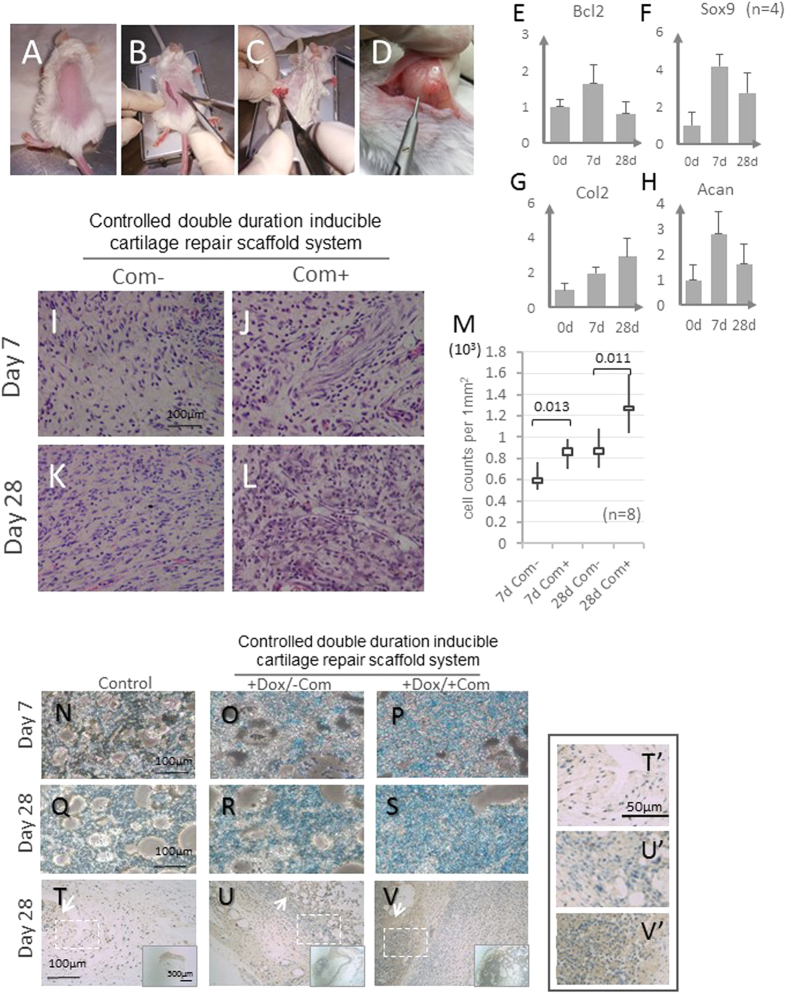
Evaluation of the system in a mouse subcutaneous implantation model. (**A**–**D**) Procedure for subcutaneous implantation model experiments. (**E**–**H**) mRNA levels of Bcl2 and chondrogenesis marker genes (Sox9, Col2 and Acan) on day 0, after one week and 4 weeks; n = 4; (**I**–**L**) H&E staining for general histological observation on day 7 and day 28; (**M**) Statistical analysis of cell numbers in H&E section staining in indicated groups; n = 8. (**N**–**S**) Alcian blue staining for general histological observation on day 7 and day 28. (**T**–**V**) Type 2 collagen immunohistochemistry in indicated groups. (**T**’–**V’**) higher magnification images cropped from dashed box areas in (**T**–**V**) respectively). White arrow, selective positive collagen II staining. The scale bars for H&E and Alcian blue staining are as indicated; Dox: Doxycycline; Com: coumermycin.

We subsequently evaluated our system’s performance in the mouse subcutaneous implantation model (Fig. 5A–D). Upon surgery, two batches of mice were sacrificed after 7 days and 28 days. Gene expression analysis indicated that anti-apoptotic Bcl-2 levels were elevated in the group sacrificed after 7 days and were at a basal level in the group sacrificed on day 28, suggesting that the controlled short term inducible expression also holds for the *in vivo* situation (Fig. 5E). On the other hand, the upregulation of chondrogenic markers was maintained in both the day 7 and day 28 groups (Fig. 5F–H), which is consistent with the rational design and previous *in vitro* experiments. To further evaluate the effectiveness of the system in the mouse subcutaneous implantation model, we used HE staining and alcian blue staining of section samples of the implants to inspect for chondrogenic changes histologically. Notably, the HE staining results indicated an increase in viable cells that were positively stained under short-term coumermycin induction conditions for both day 7 and day 28 groups, indicating a lasting impact of the short-term anti-apoptotic intervention (Fig. 5I–L,M for statistic quantification). On the other hand, implanted cells under dox induction conditions either with or without initial coumermycin induction accumulated more extracellular matrix as indicated by alcian blue staining (Fig. 5N–S). However, even more acidic matrix polysaccharides were present in the group with both induction systems present (Fig. 5P,S), again corroborating the contribution of the short-term anti-apoptotic induction to overall chondrogenic efficiency. At day 28 after implantation, we further analyzed the type II collagen secretion through Col2A1 immunohistochemistry staining of the implant sections; As expected, moderately more cartilage type II collagen was accumulated in the implant group with the Sox9 long-term induction system (Fig. 5T–V,T’–V’ for higher magnification), and even more staining was observed in groups with both induction systems, possibly due to the improved chondrogenic cellularity (Fig. 5V,V’). Collectively, these results show the effectiveness and performance of the rationally designed controlled dual duration gene expression system in cartilage repair in the mouse subcutaneous implantation model.

### Evaluation of our system in an osteochondral restoration model

**Figure 6:**
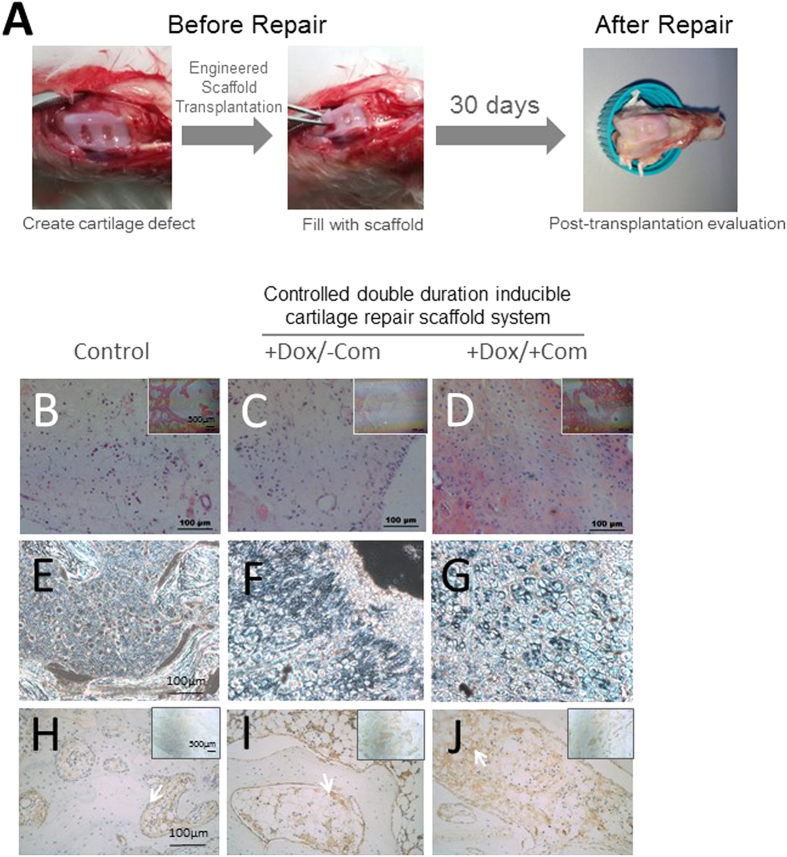
Evaluation of the system in an artificial cartilage defect osteochondral restoration model. (**A**) Procedure of making the cartilage defect model and transplantation repair. (**B**–**D**) H&E staining for general histological observation in indicated groups. (**E**–**G**) Alcian blue staining for general histological observation in indicated groups. (**H**–**J**) Type 2 collagen immunohistochemistry in indicated groups. White arrow, selective positive collagen II staining. The scale bars for H&E staining and Alcian blue staining are as indicated; Dox: Doxycycline; Com: coumermycin; n = 6.

Finally, to better simulate potential real-life applications, we tested whether our system could achieve better healing performance in a rabbit osteochondral restoration model. Full thickness cartilage defects that extend through the cartilage layer and penetrate the subchondral bone were created surgically on the femoropatellar groove of the knee joints of experimental rabbits (Fig. 6A). The gelatin sponge scaffold was implanted and fixated in the lesion using a press-fit method. After 30 days, the implants were retrieved for analysis (Fig. 6A, right). After surgery, all animals could move freely with a slightly reduced capacity of joint activity within the first 2 weeks after surgery and behaved normally thereafter. Similar to the mouse subcutaneous implantation model, HE staining identified increased cellularity in the groups with both dox and coumermycin controlled release compared with the single chondrogenic induction group, while both engineered groups out-performed the controls without any induction (Fig. 6B–D). Additionally, moderately more acid matrix polysaccharide and type II collagen secretion was observed in both groups containing the controlled double-duration inducible gene expression system (Fig. 6F,G,I,J) compared to the negative controls (Fig. 6E,H). In conclusion, these results demonstrate the conceptual feasibility of our rationally designed system in an osteochondral restoration model, closely simulating real-life applications.

## Discussion

Precisely controllable repair is one of the ultimate goals in tissue engineering research. For precise and adjustable repair procedures, gene therapy seems to be the most promising approach among existing research paradigms. Gene therapy is advantageous as it is a fast and effective way to regulate seed cells, which makes it a good supplementary approach in chondrocyte tissue engineering together with biomaterial scaffold implantation. However, recent research also showed that continuous overexpression of genes that bring short-term regenerative benefits can lead to significant long-term damage. For example, in the case of Sox9, a transcription factor that is widely used to induce early chondrogenesis^30^,^31^, uncontrolled continuous overexpression may actually inhibit later chondrogenesis^32^. Additionally, continuous overexpression of anti-apoptotic genes such as Bcl-2 employed in this study, may potentially lead to cancerous proliferation in the injured area. To avoid these predictable defects, which can be caused by gene overexpression, duration-controlled inducible expression seemed to be a good choice.

For long-term stable regulation of artificial implants, a suitable regulation strategy for *in vivo* repair must be chosen carefully to meet diverse needs. A good inducible system should be considered both safe and convenient. A tetracycline inducible system is possibly the most widely used inducible system in mammalian cell experiments due to its simple and convenient adaptability both for *in vitro*^33^ and *in vivo*^34^,^35^,^36^ research and even clinical purposes^34^. In parallel, coumermycin is also applied as an inducible system in mammalian cells because of its safety and efficacy both *in vitro* and *in vivo*^37^. In our experiment we found these two systems could even work well in combination. A tetracycline inducible system was chosen for long-term induction because of its adaptability for oral administration in any future implementations, allowing for prolonged treatment or increase of the drug dose^38^.

On the other hand, we further integrated the genetically engineered cells into the biomaterials scaffold both to provide mechanical properties^39^,^40^,^41^ and to support the controlled delivery of inducible molecules. Various materials have been applied for scaffolds in cartilage tissue engineering including gelatin^42^, polylactic acid^43^ and polyhydroxyalkanoates^23^. However, the combination of an inducible system with a biomaterials scaffold to meet *in vivo* requirements needs to take into account the duration of degradation, cell compatibility and the adsorption compatibility with both coumermycin and microsphere packaged doxycycline. Based on our preliminary tests, the gelatin sponge is compatible with both our cartilage tissue contexts^42^ and the controlled-release system payloads.

For sufficient evaluation of the controlled double-duration inducible gene expression system, two *in vivo* animal models were employed using surgical experiments, a subcutaneous implantation^22^ for preliminary evaluation of biocompatibility of the overall system *in vivo*, and a short term cartilage defect implantation model^23^ for a comprehensive evaluation of repair feasibility of a lesion quite similar to real-life situations. The results comprehensively demonstrated that cell cellularity and matrix secretion were enhanced by our inducible system as anticipated in animal models. However, although these outcomes might be closely associated with the controlled expression of anti-apoptotic gene and chondrogenic genes, more comprehensive evaluations in the future, including cell lineage tracing and a non-invasive fluorescent monitoring system, should be used to explain the background of the observed increase in cellularity and matrix secretion after introducing our system before testing the effectiveness of our system in real applications of repair can be justified. In addition, the repair effect of the dual-induction system carrying cartilage-seeding cells might be further comprehensively analyzed for a longer period. *In vivo* cartilage repair that lasts for a half a year or longer will convincingly show its capacity for repair, and may also reveal possible limitations for long-term repair, such as possible side effects of leaky expression of both anti-apoptotic gene and chondrogenic regulators in the long-term.

Collectively, it was surmised that long-term inducible expression of Sox9 would better achieve chondrogenic repair effects when combined with a short-term anti-apoptotic enhancement in tissue engineering seeding cells with our competent controlled double-duration inducible gene expression system. This type of designed time-course gene therapy may also lead to novel insights for other tissue engineering work in the future.

## Conclusion

In the present study we designed an integrative controllable regulation system for cartilage defect repairs. Two suitable inducible systems, a Tet-inducible system and a Com-inducible system were chosen and tested because both of these systems can work well *in vitro* and *in vivo*. Bcl-2 and Sox9 were chosen as functional genes because they show good anti-apoptotic and chondrogenic effects in MSC cells. A gelatin sponge was used as biomaterial scaffold, and C3H10T1/2 cells were used as seeding cells for cartilage repair. Direct adsorption and a PLGA microsphere slow release system were used for short-term and long-term gene induction, and both showed good release curves and induction effects. The whole system was tested *in vitro* and implanted into mice subcutaneously and into artificial cartilage defects in rabbits. In both *in vitro* and *in vivo* experiments, the system showed good properties with increased cell cellularity and enhanced chondrogenic matrix secretion. These *in vitro* and *in vivo* repair results collectively confirmed that such a controlled double-duration inducible gene expression system is a promising approach in cartilage defect repair. Furthermore, the approach and modular construction method may also be used to fit different needs in other tissue engineering applications.

## Additional Information

**How to cite this article**: Ma, Y. *et al.* A controlled double-duration inducible gene expression system for cartilage tissue engineering. *Sci. Rep.* **6**, 26617; doi: 10.1038/srep26617 (2016).

## Supplementary Material

Supplementary Information

## Figures and Tables

**Table 1:** Experimental design for subcutaneous implantation model.

Group treatment	Expected gene expression	Numbers of subcutaneous implant
Control	−sox9/−bcl2	4 (1week)/4 (4weeks)
+dox/−com	+sox9/−bcl2	4 (1week)/4 (4weeks)
+dox/+com	+sox9/+bcl2	4 (1week)/4 (4weeks)

**Table 2:** Experimental design for osteochondral restoration model.

Group treatment	Expected gene expression	Numbers of cartilage defects
Control	−sox9/−bcl2	6
+dox/−com	+sox9/−bcl2	6
+dox/+com	+sox9/+bcl2	6
