# Supplementary material for: A controlled double-duration inducible gene expression system for cartilage tissue engineering
Source: Sci Rep. 2016 May 25;6:26617. doi: 10.1038/srep26617 (PMC4879534; doi:10.1038/srep26617)
Supplement: Supplementary Information [file srep26617-s1.pdf]

Supplementary Material for

A controlled double duration inducible gene expression system for cartilage tissue engineering

Ying Ma[1,2,¶] Junxiang Li[1,2,¶], Yi Yao[1,2], Rui Wang[1,2], Daixu Wei[1,2], Qiong Wu[1,2,*]

1. MOE Key Laboratory of Bioinformatics, Center for Synthetic and Systems Biology, Tsinghua University, Beijing, China.

2. School of Life Sciences, Tsinghua University, Beijing, China.

¶: These authors contributed equally to this work.

*: Corresponding author, E-mail: <wuqiong@tsinghua.edu.cn>

Supplementary data legends:

Figure S1: The combination of two systems showed no cross-reactivity in induction.
(A1, A2): +10μg/ml dox; (B1, B2): +10nM coumermycin; (C1, C2): +10μg/ml dox +10nM coumermycin; (A1,B1,C1): the field of vision under green fluorescence；(A2, B2, C2): the field of vision under red fluorescence (D): Quantitative analysis of fluorescent cell counts of groups A, B and C.

Table S1: Details of plasmids used in this study.

Table S2: qPCR primers for indicated genes used in this study.

Figure S1:

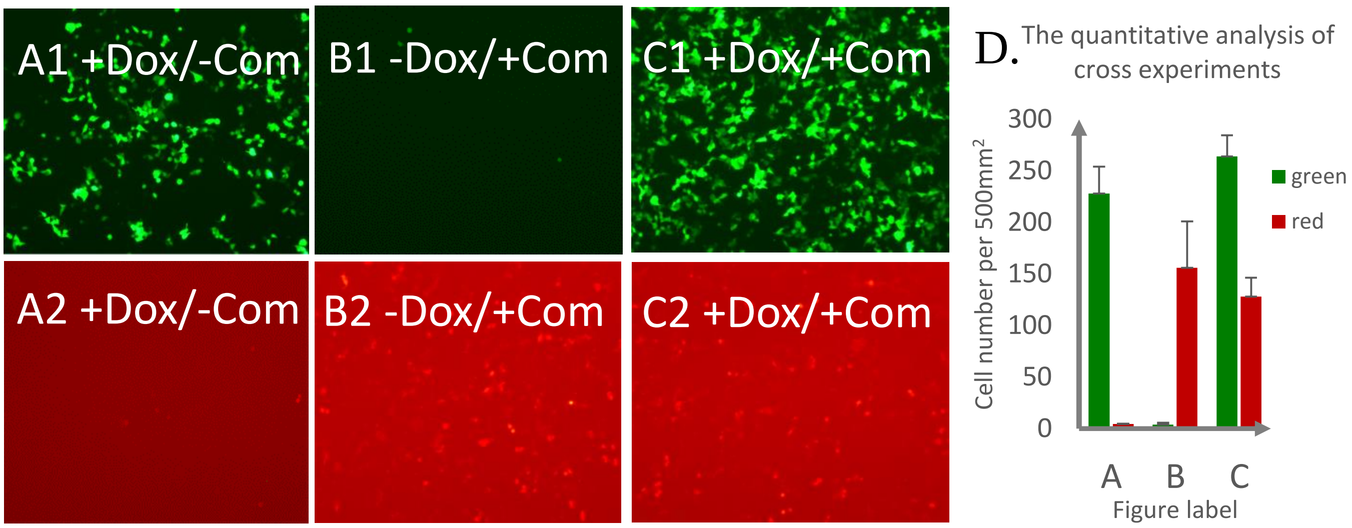
A1 +Dox/-Com
B1 -Dox/+Com
C1 +Dox/+Com
A2 +Dox/-Com
B2 -Dox/+Com
C2 +Dox/+Com
D.
The quantitative analysis of cross experiments
Cell number per 500mm^2
300
250
200
150
100
50
0
green
red
A
B
C
Figure label

Table S1:

Plasmid ID	Description	Source
EGFP-N1	EGFP expression plasmid	Clonetech
RTTA	RTTA expression plasmid	Clonetech
Ptre-EGFP	EGFP expression plasmid with Dox inducible promoter	Constructed in [22]
Ptre-Sox9	Sox9 expression plasmid with Dox inducible promoter	Constructed in [22]
pREG	REG expression plasmid	Promega
PF12K	Plasmid with Com inducible promoter	Promega
PF12K-RFP	RFP expression plasmid with Dox inducible promoter	This work
PF12K-Bcl-2	Bcl-2 expression plasmid with Dox inducible promoter	This work

Table S2:

Gene	Forward Primers	Reverse Primers
GAPDH	F:AGGTCGGTGTGAACGGATTTG	R:TGTAGACCATGTAGTTGAGGTCA
SOX9	F: CGCTCGCAATACGACTACGC	R: TAGAGCCCTGAGCCCTGGCC
Col2a	F:TGGTGGAGCAGCAAGAGCAA	R:CAGTGGACAGTAGACGGAGGAAA
Acan	F: CAGGGTTCCCAGTGTTCAGT	R: CTGCTCCCAGTCTCAACTCC
Bcl2	F: GCTACCGTCGTGACTTCGC	R: CCCCACCGAACTCAAAGAAGG
